# Clinical and radiological outcome following trauma-related reverse shoulder arthroplasty

**DOI:** 10.1007/s00402-024-05395-2

**Published:** 2024-06-07

**Authors:** Cornelius Sebastian Fischer, Christian Lohr, Patrick Ziegler, Daniel Schüll, Felix Christioph Finger, Tina Histing, Moritz Herbst, Philipp Hemmann

**Affiliations:** 1 Department of Traumatology and Reconstructive Surgery, BG Unfallklinik Tübingen, Eberhard Karls University Tübingen, Schnarrenbergstraße 95, 72076 Tübingen, Germany; 2 Department of Orthopaedics and Trauma Surgery, Klinik Gut, Via Arona 34, St. Moritz, 7500 Switzerland

**Keywords:** Reverse shoulder arthroplasty, Primary fracture RSA, Secondary RSA, Tuberosity healing, Clinical outcome

## Abstract

**Background:**

Reverse shoulder arthroplasty (RSA) is a frequently used therapy for complex proximal humeral fractures and posttraumatic disorders. The present study’s purpose was to assess the clinical and radiological outcome of primary and secondary RSA, and to analyze the impact of refixation of the greater tuberosity (GT).

**Patients and methods:**

28 patients with primary fracture RSA and 18 patients with RSA due to posttraumatic disorders were examined with a mean clinical follow-up of 2.5 ± 1.73 years. Operative details and radiographs were retrospectively reviewed. Additional analyses were performed for healed and non-healed GT in primary RSA.

**Results:**

Patients with fracture RSA had higher Constant-Murley score (CMS) than secondary RSA without reaching significance (*p* = 0.104). No significant difference was present for the quality of life measured by the Short Form 36 (SF 36) and the range of motion. In primary RSA, 78.6% GT healed anatomically. Compared to non-healed GT, patients with healed GT had a significantly higher CMS (*p* = 0.011), external rotation (*p* = 0.026) and forward flexion (*p* = 0.083), whereas DASH score was lower without a significant difference (*p* = 0.268). SF 36 showed no significant difference. Patients with healed GT had a more neutral glenoid version (*p* = 0.009).

**Conclusion:**

Superior range of motion and clinical outcome scores were present for anatomically healed GT. Therefore, refixation of the tuberosities is recommended. Secondary RSA can result in inferior results compared to primary RSA, so patients need to be adequately informed.

**Supplementary Information:**

The online version contains supplementary material available at 10.1007/s00402-024-05395-2.

## Introduction

Proximal humeral fractures (PHF) are among the most common fractures in elderly patients [1, 2]. Due to demographic changes, the incidence of PHF will further increase [1]. The severity of fractures in geriatric patients increases the necessity to use joint replacement instead of reconstructive procedures. Especially elderly patients should undergo single surgery due to comorbidities and to minimize the risk of complications, e.g. avascular humeral head necrosis or implant failure.

Reverse shoulder arthroplasty (RSA) became one of the most efficient surgical options when treating complex PHF [3–5]. The RSA design medializes and lowers the center of rotation [2]. Consequently, the deltoid muscle plays a more important role in shoulder function than the rotator cuff. Cuff tear arthropathy is an indication for RSA in the presence of corresponding symptoms. Other indications for RSA, like posttraumatic arthritis, primary osteoarthritis or rheumatoid arthritis have been investigated in several studies [6, 7]. Compared to primary trauma RSA, inferior results [8–11] and higher revisions rates [10, 12, 13] are reported for secondary RSA.

For primary RSA, there is an ongoing discussion about the benefits of tuberosity fixation [2]. Missing infraspinatus and teres minor muscles are limiting external rotation and therefore, current treatment options prefer tuberosity refixation [2]. Studies reported a wide range of tuberosity healing rate [14]. Anatomic healing of the greater tuberosity (GT) was associated with better functional outcomes such as better range of motion (ROM) and shoulder function such as lower complication rates [2, 4, 5, 15, 16]. Other studies excised the tuberosities in RSA [17, 18].

Therefore, our study aimed to evaluate the clinical and radiological outcome after RSA due to non-reconstructable PHF and RSA following posttraumatic disorders. Additionally, the study examined how the healing rate of the greater tuberosity impacts clinical outcome.

## Methods

### Design and sample

**Fig. 1:**
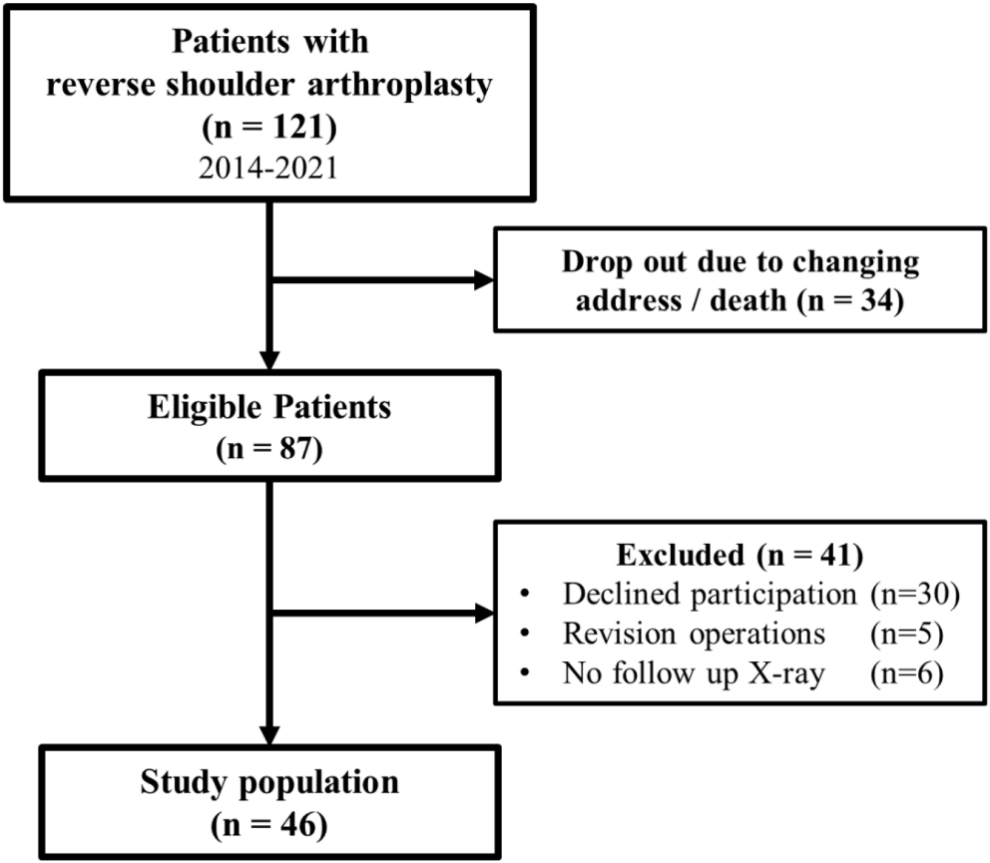
Flowchart of examined patients

In this retrospective study, all patients who received RSA due to non-reconstructable 3 and 4 -part-PHF or posttraumatic disorders in our level I trauma center between 2014 and 2021 were included. Out of 121 possible participants 34 could not be included due to missing data or death. Out of the remaining 87 eligible participants, 5 participants had to be excluded due to revision operations in other hospitals resulting in missing data. 30 declined participation due to personal reasons like high age or living far away. All remaining 52 participants were invited to an interview and physical examination. 30 patients attended in person and 22 participated via questionnaire due to personal reasons (e.g. living far away, coronavirus pandemic, etc.). 6 participants with primary RSA had to be excluded due to missing radiographic data (Fig. 1). Each participant gave written informed consent. The study was approved by the local ethics committee (project number 972/2020BO2).

### Patient-related parameters

For each participant, age and gender as well as functional scores were recorded. Every patient performed the Disabilities of the Arm, Shoulder and Hand (DASH) questionnaire, Constant-Murley-Score (CMS) as well as the Short Form 36 (SF 36) health survey. The SF 36 describes the subjective health-related quality of life and is adjusted for age, gender, and geographical differences. It consists of eight subscales. Out of those subscales two sum scores, the physical component scale (PCS) and the mental component (MCS) can be formed. PCS represents the physical state of health, MCS the mental state of health. Participants who could not be present for the examination performed the Constant-Murley-Score for self-evaluation (Böhm et al. 2004). For these patients, the strength assessment of the CMS was not assessed due to its difficult reliability. For personally attending participants, a clinical examination was conducted.

### Surgical procedure

89% of the RSH were implanted through a deltopectoral approach. In 5 cases a delta-split approach was used. 31 Aequalis Reverse Fx prosthesis (Wright Medical Group Inc., Memphis, TN, USA), 14 Delta Xtend reverse prosthesis (DePuy Synthes, Warsaw, IN, USA), both with a neck-shaft angle of 155° were implanted. One patient received a Comprehensive reverse prosthesis (Zimmer Biomet Inc., Warsaw, IN, USA) with a neck-shaft angle of 135°. For primary RSA, a transosseous fixation with fiber wire sutures was used for tuberosity fixation in 14 cases. In the other 14 cases, an additional fiber wire was passed around the prosthesis. Approach, implant and tuberosity fixation technique were chosen by the surgeons’ preference. Postoperatively, all patients wore a sling with 15° abduction in neutral rotation for 6 weeks. Passive mobilization was started on the first postoperative day. Abduction was limited to 60° for 3 weeks and to 90° for additional 3 weeks. After 6 weeks the sling was removed and active mobilization with full ROM was allowed. If the intraoperatively assessed bone quality was poor, active mobilization was delayed. Weight-bearing was restricted for 12 weeks. Return to all activities was permitted after 6 months.

### Radiographic assessment

All radiographic imaging of our institution was evaluated by a surgeon with 5 years of experience and a trained observer. Good intra - and interrater variability between these observers was determined with intraclass correlation coefficients between 0.992 and 0.906. In patients without any physical complaints, available radiographs were used without additional radiographic assessment due to ethical reasons. Fracture type was assessed on the preoperative x-ray and CT scans according to the Neer classification [19]. Additionally, the critical shoulder angle (CSA) [20] and the glenoid version [21] were determined. Regarding tuberosity healing, only the greater tuberosity was assessed, because the evaluation of the lesser tuberosity is not reliable enough [22].In the last performed radiograph in our institution the healing of the greater tuberosity (GT) was classified into anatomically healed (complete, partially resorbed or incompletely healed) or not healed (dislocated or completely resorbed). Complete anatomical healing was defined as visible GT in all postoperative x-rays in AP view without any migration or resorption of GT. If the GT was smaller or more radiolucent then in the postoperative imaging, it was defined as partially resorbed. Incompletely healed was defined as steady GT in multiple postoperative x-rays without cranialization above the upper end of the polyethylene and without a bony connection to the humerus.

Additionally, scapular notching was assessed in 4 grades according to the Sirveaux classification [23]. The peg-glenoid rim distance (PGRD) was measured by the distance between the inferior glenoid rim and the upper margin of the central peg or screw [24]. Moreover, the overhang of the glenosphere on the inferior scapular neck was measured [25]. Humeral loosening was assessed using the classification of Melis et al. [26].

### Statistics

Descriptive statistics such as mean values, standard deviations (SD), ranges, medians and percentiles were used to describe the sample. Spearman-Correlation was used for numerical continuous variables. The Mann-Whitney-U-test and Kruskal-Wallis-test were used to examine numerical data. Categorical data was examined with Chi2-test (Pearson-Chi2). If the expected frequency was less than 5, the data was analyzed with the Fisher-Freeman-Halton-exact-test. The paired T-test was used for the evaluation of changes within the same subjects. A p-value of < 0.05 was considered as statistically significant. The statistical analysis was performed using SPSS version 28 for Windows (Armonk, NY; IBM Corp.).

## Results

76% of the participants were females while the mean age was 72 ± 10.7 years. The mean clinical follow-up was 2.5 ± 1.73 years, while the mean radiological follow-up was 2 ± 1.3 years. Out of the 28 primary fracture RSA, 21.4% were due to non-reconstrucTable 3-part fractures, while the remaining were 4-part fractures. The secondary RSA group (*n* = 18) consisted of 9 participants with severe posttraumatic arthritis, 4 patients with posttraumatic humeral head necrosis, 1 patient with resection of the humeral head due to posttraumatic infection, 3 patients with chronic instability following traumatic shoulder luxation and one patient with conversion from anatomical fracture prosthesis to RSA. All those patients underwent multiple surgeries prior to the RSA implantation. Additional information on patient’s demographics and surgery specifics are provided in Table 1.

**Table 1:** Study cohort characteristics

n	Total	Fracture RSA	Secondary RSA
	46	28	18
Age [years]	72.0 (10.7) [39–89]	76.4 (8.5) [59–89]	65.2 (10.4) [39–78]
Female [n]	35 (76%)	23 (82%)	12 (66.7%)
Right-handed [n]	42 (91%)	24 (85.7%)	18 (100%)
Right Side of RSA [n]	24 (52%)	15 (54%)	9 (50%)
Deltopectoral Approach [n]	41 (89.1%)	25 (89.3%)	16 (88.9%)

Data are presented as mean (SD) [range]

The radiological assessment showed only one RSA with signs of humeral loosening. Overall, the CSA changed significantly pre- and postoperatively for fracture RSA (*p* = 0.003). Smaller non-significant changes were present in secondary RSA implantations (*p* = 0.415). No significant difference in glenoid version was present between fracture and secondary RSA (*p* = 0.086). 96.4% fracture RSA and 88.9% secondary RSA showed no signs of notching. Detailed information is presented in Table 2. Patients with signs of scapular notching had a more retroverted glenoid (-0.73° vs. -11.2°, *p* = 0.003). However, only 3 patients had minor signs of notching.

**Table 2:** Radiological parameters

*n*	Total	Fracture RSA	Secondary RSA	p*
	46	28	18	
Preoperative CSA [degree]	33.3 (5.7) [22.1–46]	33.9 (6.1) [23.6–46]	32.3 (5.2) [22.1–42.5]	0.375
Postoperative CSA [degree]	29.9 (7.3) [12.3–43.9]	27.3 (5.9) [17-41.2]	31.6 (8.8) [12.3–43.9]	0.096
Glenoid version [degree]	-1.5 (8.1) [-28.6–12.7] ^#^	0.4 (7.5) [-17.8 -12.7] ^#^	-4.1 (8.5) [-28.6-7.9]	0.086
PGRD [mm]	18.4 (2.6) [11.7–23.5] ^y^	17.6 (2.4) [11.7–22] ^y^	19.6 (2.5) [14.4–23.5]	0.008
Glenosphere overhang [mm]	5.7 (2.3) [0.0-10.9] ^y^	6 (2.3) [1.9–10.9] ^y^	5.2 (2.4) [0.0-9.3]	0.431
Notching Grade 1 [n]	3 (6.5%)	1 (3.6%)	2 (11.1%)	0.336°

Data are presented as mean (SD) [range], CSA = critical shoulder angle, PGRD = peg-glenoid rim distance

* Mann-Whitney-U-test

° Fisher-exact-test

# 2 patients missing, ^y^1 patient missing

The CMS showed higher values in patients with fracture RSA. Accordingly, the DASH showed a lower functional impairment. However, the quality of life measured by SF 36 was not significantly different between fracture and secondary RSA (Table 3). No significant differences in ROM were present between patients with fracture RSA and secondary RSA for forward flexion (*p* = 0.476), abduction (*p* = 0.088), external rotation (*p* = 0.907) and internal rotation (*p* = 0.748).

**Table 3:** Functional scores and quality of life for fracture and secondary RSA

*n*	Total	Fracture RSA	Secondary RSA	p*
	46	28	18	
CMS^#^	43.1 (15.2) [2–69]^x^	46.4 (12.5) [26–65]^x^	38.4 (17.7) [2–69]	0.104
DASH	34.7 (20.9) [5-73.3]^x^	34.2 (19.7) [5.2–73.3]^x^	35.5 (23.1) [5-73.3]	0.950
physical component scale (SF36)	34.6 (10.4) [12.8–59.5]^x^	33.1 (9.9) [18.7–59.5]^y^	37.2 (10.9) [12.8–57.9]^y^	0.085
mental component scale (SF36)	49.7 (12.1) [18.4–66.9]^x^	49.6 (11.8) [25.1–66.9]^y^	49.9 (12.9) [18.4–63.6]^y^	0.86

Data are presented as mean (SD) [range], 2 patients missing due to incomplete scores, CMS = Constant-Murley-Score, DASH = Disabilities of Arm, Shoulder and Hand score, SF 36 = Short Form 36 health survey

^x^ 2 patients missing, ^y^ 1 patient missing

^#^max. points 75, due to missing information on arm strength

* Mann-Whitney-U-test

There was no significant association between the assessed radiological parameters and postoperative function and quality of life (Table 4).

**Table 4:** Associations between radiological parameters and range of motion, functional scores and quality of life

*N*	Glenoid version	Postoperative CSA	PGRD	Glenosphere Overhang
	44	46	45	45
CMS^#^	0.616^y^	0.264^x^	0.049^x^	0.086^x^
DASH	0.198^x^	0.746^z^	0.156^z^	0.212^z^
physical component scale (SF36)	0.568^x^	0.487^x^	0.552^x^	0.389^x^
mental component scale (SF36)	0.599^x^	0.984^x^	0.823^x^	0.330^x^
Forward flexion*	0.610	0.871	0.688	0.311
Abduction*	0.760	0.242	0.776	0.605
Internal Rotation*	0.390	0.395	0.092	0.105
External Rotation*	0.888	0.561	0.745	0.931

^#^max. points 75, due to missing information on arm strength

^x^ 2 patients missing, ^y^ 1 patient missing, ^z^ 3 patients missing

CSA = critical shoulder angle, PGRD = peg-glenoid rim distance

* Kruskall-Wallis-test

In the last performed X-ray, of 22 healed greater tuberosities 17 were anatomical without any signs of resorption, 3 GT were anatomically healed with partially resorbed areas, 2 GT were in correct position, but without complete bony connection to the humeral shaft. The non-healed group consisted of 4 dislocated GT and 2 completely resorbed GT. 50% of all healed and non-healed GT had an additional fiber wire around the prosthesis for refixation. Patients with healed GT had a significantly higher function in the CMS (*p* = 0.011) and lower, but statistically not significant, impairment in the DASH score (*p* = 0.268). No significant difference in quality of life (SF36) was present for patients without sufficient GT healing (Table 5). Compared to secondary RSA, patients with healed GT had significantly better CMS (*p* = 0.026) while DASH (*p* = 0.752) and SF36 (mental component scale: *p* = 0.900, physical component scale: *p* = 0.110) were not significant.

**Table 5:** Functional scores and quality of life for patients with Healed and Non-Healed Greater Tuberosity following fracture RSA

*n*	Healed GT	Non-Healed GT	*p*
	22	6	
CMS^#^	49.8 (11) [26–65]^x^	35.2 (11.2) [26–55]	0.011
DASH	32.5 (20.9) [5.2–73.3] ^x^	40 (15.1) [23.3–63.3]	0.268
physical component scale (SF36)	33.2 (10.7) [18.7–59.5]	32.4 (6.2) [22.4–38.5]^y^	0.928
mental component scale (SF36)	50.1 (11.5) [25.1–66.9]	47.0 (14.6) [31.1–63.2]^y^	0.880

Data are presented as mean (SD) [range], CMS = Constant-Murley-Score, DASH = Disabilities of Arm, Shoulder and Hand score, SF 36 = Short Form 36 health survey

^#^max. points 75, due to missing information on arm strength

**Fig. 2:**
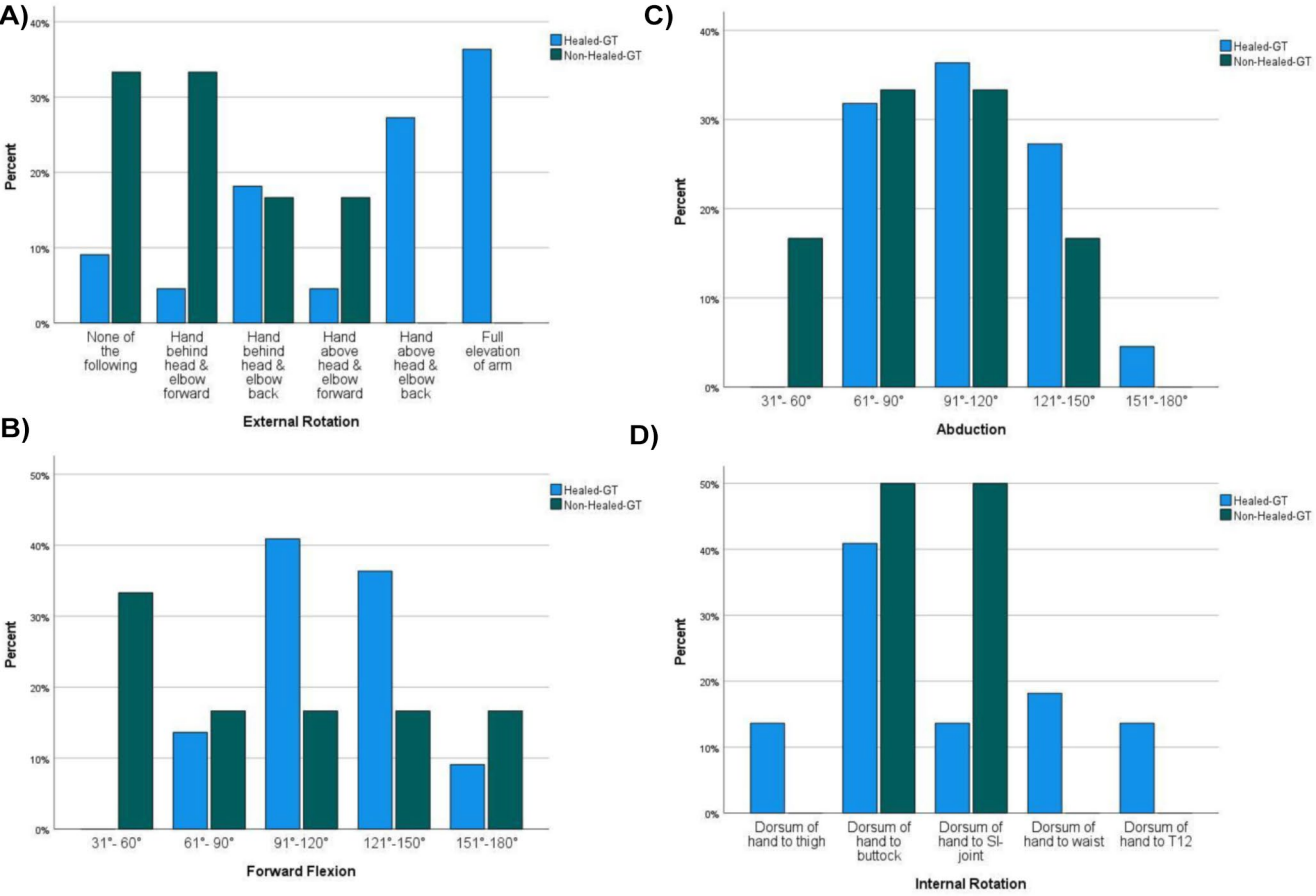
(**A**-**D**) Range of motion on CMS divided into healed and non-healed tuberosity

Patients with healed GT had higher values in ROM (Fig. 2). External rotation was significantly higher for patients with healed GT (*p* = 0.026), forward flexion (*p* = 0.083), abduction (*p* = 0.546) and internal rotation (*p* = 0.366) did not show statistical significance.

Regarding radiological parameters, patients with healed GT had a more neutral glenoid (-1.5 ± 7.1 vs. 6.9 ± 5, *p* = 0.009). No significant associations were present for CSA (*p* = 0.723), PGRD (*p* = 0.977), glenosphere overhang (*p* = 0.755) or notching (*p* = 1.0). No significant differences between refixation technique (*p* = 0.479), prosthesis design (*p* = 0.886) or brand (*p* = 0.484) in regard to clinical outcome were present.

## Discussion

Implantation of RSA due to severe trauma is an increasingly used therapy in the last years [27] as well as in secondary posttraumatic disorders. However, RSA implantation can have a major impact on patients’ quality of life. Therefore, understanding the outcome is crucial in determining the success of the procedure and guiding further strategies to optimize the treatment and therefore the patient’s quality of life and functionality.

For primary trauma RSA, several influencing factors have lately been discussed, such as prosthesis design with different humeral inclination (135° vs. 155°) [28] and fracture specific stems [4]. Another controversial discussion is about potential benefits of tuberosity fixation. Cazeneuve et al. recommended to not reattach or to excise the tuberosities to prevent impingement [17], while others described an inferior clinical outcome [15] with a higher rate of instability [22] and higher complication rates in patients without anatomically healed GT [4, 5, 29].

Nonetheless, recent research indicates that anatomic healing of the GT leads to improvements in ROM. The finite element analysis of Sabesan et al. assumed no impact of GT healing for abduction and forward flexion while external rotation was affected [30]. Clinical studies revealed various results. Some studies did not show any improvement in ROM for reconstructed tuberosities [15, 31]. However in the work of Marin et al., most reconstructed tuberosities were healed in malposition or were reabsorbed [15]. Several studies reported improved forward flexion [4, 5, 14, 32, 33] and/or external rotation [4, 5, 14, 32–35] as well as abduction [33]. Recent reviews concluded that patients with healed GT following fracture RSA exhibited better forward flexion, abduction and external rotation [16, 28]. The present study detected significantly higher values for external rotation (*p* = 0.026), while forward flexion did not reach significance (*p* = 0.083).

Regarding clinical outcome scores inconsistent results have been reported. Some studies observed better CMS in patients with healed GT [4, 14, 22, 33, 34], others did not confirm this [15, 31, 35–37]. In DASH, various results are present as well [15, 34, 35]. In the present study, significantly higher CMS values (*p* = 0.011) and lower, but statistically not significant, impairment in the DASH score (*p* = 0.268) was observed for patients with healed GT. Torrens et al. documented an influence of age on the CMS and that comorbidities interfered with GT healing [36]. Additionally, recent studies showed that malposition, reabsorption [15] or migration [2] of the tuberosities can even lead to inferior results, whereas Gallinet et al. described superior clinical outcomes when the tuberosities did not heal in an anatomical position [5]. Since the present results and many other studies show better functional outcome for healed GT, refixation of the tuberosities to enable anatomic healing seems advisable. Since primary trauma RSA is a frequently used therapy especially for elderly patients with lower functional demand, additional scores for quality of life (e.g. SF36) might be necessary to assess the outcome and satisfaction of these patients. Unfortunately, the questionnaires include many items which target the function of the lower extremity. This could distort the results if the outcome following surgery on the upper limp is under evaluation. However, our sample did not show any difference between healed and non-healed GT in reported quality of life (SF 36). Therefore, we agree with Sabesan et al., who concluded that good results can be expected for patients even with poor GT healing [30].

Like the present sample, only small sample sizes are investigated in current literature about RSA in posttraumatic disorders. Present data support that RSA is a valuable tool for fracture sequeale [38, 39]. However, compared to primary RSA, inferior results [8–11] and higher revision rates [10, 12, 13] are reported. Kim et al. documented the lowest subjective satisfaction for patients with posttraumatic arthritis compared to other etiologies [6]. Several authors described inferior ROM in secondary RSA, while shoulder scores were not always significantly altered in comparison with primary RSA [8, 12, 13, 39]. Shannon et al. concluded that RSA after failed osteosynthesis results in comparable functional outcomes [40]. In the present study, inferior results were present for CMS and DASH without reaching significance. In comparison to the healed GT group, the inferior CMS for secondary RSA was significant. As expected, patients with secondary RSA were younger than patients with primary trauma RSA, since reconstructive surgery is often preferred for younger patients. The younger age might also explain the higher values of the physical component scale of the SF36 for patients with secondary RSA, because several questions about mobility are part of the SF36. Overall, we agree that results for secondary RSA are less predictable, and patients should therefore be adequately informed.

In the present sample, no significant associations between clinical outcome and radiological parameters were present. There was a notching rate of 6,5%, which is relatively low compared to previously reported rates between 8% [33] and 88% [26]. A possible reason might be the inferior glenosphere overhang, which is associated with lower notching rates [9, 25].

This study has some limitations. First, the CMS was assessed without strength scale. Boehm et al. described a good correlation for their strength assessment, but the acquisition of this correlation seemed to be challenging [41]. The French “Auto-Constant” showed a less accurate evaluation for strength as well [42]. Another problem is the position of the strength assessment as Ziegler et al. determined [43]. Therefore, we resigned from the strength assessment during self-assessment of the CMS, because the acquisition of reliable values seems to be problematic. Secondly, Uzer et al. described better clinical outcomes and healing rates of the tuberosities following autograft augmentation of the fixated tuberosity [44], while other authors determined superior results for tuberosity healing for a RSA design with a humeral inclination of 135° compared to an inclination of 155° [28, 29, 45]. Different results regarding the influence of fracture specific stems and cementation on healing rates of the tuberosity are present as well [4, 46–48]. However, different models of RSA with mostly cemented fracture specific stems were used in the present sample. Another limitation might be that the fixation technique of the tuberosity was performed according to the surgeon’s preference. Various fixation techniques are described in present research [3, 49, 50]. Even tensioning devices were proposed recently [51]. However, our tuberosity healing rate of 78,6% seems to be above the calculated mean of 70.5% by the review of Jain et al. [16]. Another limitation is that, due to the small sample groups, adequately powered calculations are difficult. Additionally, the retrospective approach does not allow cause-effect-relationships. A further limitation might be the possible resorption of the GT in patients with shorter follow-up. This study has a relatively short period of follow-up. Several patients received out-clinic follow up radiographs, so additional radiographs were not indicated for satisfied patients due to ethical reasons. However, long term follow-up and further studies with large sample sizes and standardized design are needed.

In conclusion, the present study detected a better ROM and clinical outcome for healed GT. Therefore, patients might benefit from correct healing of the tuberosities, so anatomic refixation should be performed. Secondary RSA resulted in inferior results without reaching significance. However, prior to the implantation of secondary RSA, adequate information on less predictable clinical results for patients are advisable.

### Electronic supplementary material

Below is the link to the electronic supplementary material.

Supplementary Material 1

Supplementary Material 2

Supplementary Material 3

Supplementary Material 4

Supplementary Material 5

Supplementary Material 6

Supplementary Material 7

Supplementary Material 8
